# Correction: Neural mechanisms of psychedelic visual imagery

**DOI:** 10.1038/s41380-024-02818-9

**Published:** 2024-11-21

**Authors:** Devon Stoliker, Katrin H. Preller, Leonardo Novelli, Alan Anticevic, Gary F. Egan, Franz X. Vollenweider, Adeel Razi

**Affiliations:** 1 Turner Institute for Brain and Mental Health, School of Psychological Sciences, Monash University, Clayton, VIC Australia; 2 Department of Psychiatry, Psychotherapy & Psychosomatics, University Hospital for Psychiatry, Zurich, Switzerland; 3 Department of Psychiatry, Yale University School of Medicine, New Haven, CT USA; 4 Monash Biomedical Imaging, Monash University, Clayton, VIC Australia; 5 Wellcome Centre for Human Neuroimaging, UCL, London, UK; 6 CIFAR Azrieli Global Scholars Program, CIFAR, Toronto, ON Canada

**Keywords:** Neuroscience, Psychiatric disorders, Psychology, Biochemistry

Correction to: *Molecular Psychiatry* 10.1038/s41380-024-02632-3, published online 11 June 2024

In the sentence beginning ‘Connectivity from the EVA follows two broad pathways. The dorsal pathway is connected with the fusiform gyrus (FG), which is part of the inferior temporal cortex that functions to recognise objects and faces [45]. The ventral pathway from the EVA is connected to the intraparietal sulcus (IPS), which, along with the inferior frontal gyrus (IFG), is involved in cognitive aspects of imagery.’ the should have read ‘Connectivity from the early visual areas (EVA) follows two broad pathways. The dorsal pathway projects to the intraparietal sulcus (IPS), which plays a role in the cognitive aspects of visual imagery and spatial processing. The ventral pathway connects to the fusiform gyrus (FG), part of the inferior temporal cortex involved in object and face recognition [45]. Both pathways interact with the inferior frontal gyrus (IFG), which integrates higher cognitive functions like attention and working memory, and can modulate both spatial and object-based visual information.’

In the sentence beginning “The agonism of psychedelic molecules binding to the 5-HT2AR...,” the original citation for Burt et al. (2021) has been reformatted to a numbered reference. In the sentence beginning “Similar findings are associated with eyes-closed visual alterations under the psychedelic N,N-Dimethyltryptamine (DMT)...,” the original citation for Timmermann et al. (2019) has been reformatted to a numbered reference.

The original article has been corrected.

